# Tannin in Tea

**Published:** 1893-03

**Authors:** 


### TANNIN IN TEA.

Some examples which have been forwarded to us, says the *British Medical Journal*, of the results of analyses for tannin and theine in tea indicate considerable variations in the amount of tannin, according to the quality of the tea and the state of growth at which it is picked. In some blends of China teas the percentage of tannin extracted by infusion for thirty minutes was 7.44; theine, 3.11; and a similar result was given in the examination of the finest Moying; while, on the other hand, with fine Assam tea a percentage of 17.73 of tannin by weight was extracted after infusion for fifteen minutes, and two blends of Assam and Ceylon tea, gave, respectively, 8.91 and 10.26 of tannin. On the whole, it is probable that the Indian teas are much more heavily loaded with tannin than the China and Japan teas. Moreover, the common method of prolonged infusion in boiling water is well calculated to extract the tannin, while it dissipates the flavor of the tea.

To be drunk reasonably, tea should not be infused for more than a minute, and with water of which the temperature does not exceed 170° F. It should be taken without sugar or milk, which would drown the flavor of the delicate and aromatic infusion thus obtained. This, at least is how the tea is drunk both in China and Japan, whence we have borrowed the use of it. With our European method of prolonged infusion in boiling water we destroy all the best flavor of the tea, and we extract such heavy proportions of tannin as to cultivate indigestion as the result of tea drinking. Indigestion is unknown among tea drinkers in the East, and it is in all probability only the result of defective use of the leaf.—*Scientific American.*

